# Early‐Onset Movement Disorder Syndrome Caused by Biallelic Variants in *PDE1B* Encoding Phosphodiesterase 1B

**DOI:** 10.1002/mds.30249

**Published:** 2025-06-10

**Authors:** Tomer Poleg, Noam Hadar, Eyal Kristal, Nicola Y. Roberts, Vadim Dolgin, Ilana Aminov, Amit Safran, Nadav Agam, Matan Jean, Ofek Freund, Eamonn G. Sheridan, James A. Poulter, Michelle L. Thompson, Yusra Algoos, Salma Al‐Qahtani, Lama AlAbdi, Sateesh Maddirevula, Verity Hartill, Henry Houlden, Reza Maroofian, Amit Nahum, Ohad S. Birk

**Affiliations:** ^1^ Faculty of Health Sciences Ben‐Gurion University of the Negev Be’er Sheva Israel; ^2^ Pediatric Ambulatory Unit Soroka University Medical Center Be’er Sheva Israel; ^3^ Leeds Institute of Medical Research, University of Leeds Leeds UK; ^4^ HudsonAlpha Institute of Biotechnology Huntsville Alabama USA; ^5^ Department of Translational Genomics Center for Genomic Medicine, King Faisal Specialist Hospital and Research Centre Riyadh Saudi Arabia; ^6^ Movement Disorders Program Neuroscience Centre, King Faisal Specialist Hospital and Research Centre (KFSHRC) Riyadh Saudi Arabia; ^7^ College of Medicine Alfaisal University Riyadh Saudi Arabia; ^8^ Yorkshire Regional Genetics Service Chapel Allerton Hospital Leeds UK; ^9^ Department of Neuromuscular Disease UCL Queen Square Institute of Neurology London UK; ^10^ Pediatric Division, Kaplan Medical Center, Rehovot, Israel Affiliated to the Hebrew University of Jerusalem and Hadassah Medical School Jerusalem Israel; ^11^ Genetics Institute Soroka University Medical Center Be’er Sheva Israel; ^12^ The Danek Gertner Institute of Human Genetics, Sheba Medical Center Ramat Gan Israel

**Keywords:** *PDE1B*, movement disorder, phosphodiesterase, exome sequencing, early onset

## Abstract

**Background:**

Breakdown of cyclic adenosine monophosphate (cAMP) and cyclic guanosine monophosphate (cGMP) in basal ganglia cells through hydrolysis of diesteric bonds, primarily by PDE10A and PDE1B, is essential for normal human movement. While biallelic loss‐of‐function variants in PDE10A are known to cause hyperkinetic movement disorders, the role of PDE1B in human disease has not been characterized.

**Objectives:**

We aimed to define the phenotypic and molecular characteristics of a novel autosomal recessive disorder caused by biallelic *PDE1B* variants.

**Methods:**

Clinical phenotyping by senior geneticists and neurologists, followed by whole exome sequencing, segregation analysis (Sanger sequencing), and molecular studies, including mini‐gene splicing assays and protein studies in transfected HEK293 cells.

**Results:**

Seven affected individuals from five unrelated pedigrees presented with an apparently autosomal recessive disorder characterized by hypotonia in infancy, progressing to ataxia and dystonia in early childhood, with developmental delay and intellectual disability. Biallelic *PDE1B* variants were identified in all affected individuals: three truncating (p.Q45*, p.Q86*, p.S298Afs*6) and three splicing variants (c.594 + 2 T>G, c.735 + 5G>A, c.837‐1G>C). Functional studies confirmed that the truncating variants caused loss of the catalytic domain, resulting in truncated or absent functional protein. Splicing variants led to exon skipping, frameshifts, and catalytic domain disruption. These findings establish a causative link between biallelic *PDE1B* variants and the observed clinical phenotype.

**Conclusions:**

Biallelic loss‐of‐function variants in *PDE1B* underlie a novel early‐onset movement disorder resembling the phenotype associated with *PDE10A* deficiency. © 2025 The Author(s). *Movement Disorders* published by Wiley Periodicals LLC on behalf of International Parkinson and Movement Disorder Society.

## 1 Introduction

Cyclic nucleotide signaling plays a crucial role in the pathogenesis of movement disorders.^1^ The cyclic nucleotides, cyclic adenosine monophosphate (cAMP) and cyclic guanosine monophosphate (cGMP), act as intracellular second messengers that impact signaling in the basal ganglia circuit and affect movement control.^2^ Maintaining appropriate levels of cAMP and cGMP requires a precise balance between their synthesis and degradation. Phosphodiesterase (PDE) enzymes are responsible for the breakdown of cAMP and cGMP by hydrolyzing phosphodiester bonds, thereby directly regulating the intracellular levels of these second messengers.^3^

Extensive research over the years has suggested that alterations in PDE expression and function, and subsequent variations in cyclic nucleotide levels and their downstream targets, might contribute to several movement disorders such as Huntington's disease (HD)^4^, ^5^ and Parkinson's disease (PD).^5^, ^6^ Furthermore, mutations in several PDE genes, including *PDE8B*,^7^, ^8^, ^9^, ^10^ *PDE2A*,^11^, ^12^ and *PDE10A*,^13^, ^14^, ^15^, ^16^, ^17^, ^18^ underlie rare genetic diseases manifested mainly by movement disorders. The PDEs most abundant in the basal ganglia are PDE10A (previously associated with infantile‐onset limb and orofacial dyskinesia; OMIM 616921) and *PDE1B*.

We here present a novel syndrome of axial hypotonia in infancy with progressive movement disorder throughout childhood that is caused by biallelic variants in *PDE1B*. Compiling data from seven patients of five unrelated kindreds, we identified six *PDE1B* variants, including three splicing, two truncating, and one frameshift variant, and demonstrated that those variants result in complete loss of functional protein.

## 2 Methods

### 2.1 Patients and Clinical Evaluation

The cohort was compiled through the collaboration of five research groups via GeneMatcher^19^ and personal communications. Clinical and genetic information was gathered with written informed consent using a standardized questionnaire. The study received approval from the Soroka Medical Center Institutional Review Board (IRB) (Approval #5071G), the Israel Ministry of Health National Helsinki Committee (Approval #920100319), and King Faisal Specialist Hospital and Research Centre (KFSHRC) IRB RAC#2210029. Written informed consent was obtained from all participants or their legal guardians. Phenotyping was performed by senior clinical geneticists and neurologists.

### 2.2 Exome Sequencing and Variant Interpretation

Genomic DNA was extracted from whole blood samples following established protocols.^20^ Whole exome sequencing was conducted by Macrogen® using the Agilent SureSelect Exome V7 library construction enrichment kit and the Illumina NovaSeq6000 system with 150 base pair (bp) paired‐end reads. Raw data reads were aligned to the GRCh38 reference genome using BWA‐MEM,^21^ with variant calling executed by GATK 4.2.0.0. Data analysis was carried out using VARista^22^ variant analysis software. All five investigated variants were analyzed in the gnomAD database^23^ to confirm the absence of homozygous individuals for these variants among healthy populations. All genomic data are per hg38. *PDE1B* transcript sequencing data refer to NM_000924.4.

### 2.3 Splicing Assay— *PDE1B* Mini‐Gene

To generate *PDE1B* mini‐gene wild‐type (WT) plasmid, a fragment containing the entire exons 5–10 flanked by their complete endogenous introns was polymerase chain reaction (PCR)‐amplified from WT genomic DNA sample (PCR primers: forward 5′‐AGCCCAAGTTCCGAAGCATT‐3′ and reverse 5′‐AGCTACGATGAGAACCCCCA‐3′). Eventually, a full fragment of 4687 bp was subcloned into pcDNA3.1 mammalian expression vector by Gibson assembly cloning (Thermo Fisher Scientific). Mini‐gene vectors harboring each of the investigated splicing variants were generated through separate PCR amplification using primers that contained each of the studied variants (c.594 + 2 T>G; forward 5′‐ GCTTCAAGGGTGGGCAGCATCC‐3′ and reverse primer 5′‐ GCTGCCCACCCTTGAAGCGG‐3′. C.735 + 5G>A; forward 5′‐ GATGGTGGTAGATGCCCTGGAGATGAT‐3′ and reverse primer 5′‐ CAGGGCATCTACCACCATCCCTG‐3′). SH‐SY5Y cells were transfected using polyethylenimine (PEI) as a transfection reagent, transiently overexpressing in these cells either the WT vector or the splicing variant‐bearing mini‐gene vectors (c.594 + 2 T>G, c.735 + 5G>A). Total RNA was extracted using GENzol™ Tri RNA Pure Kit (Geneaid Biotech Ltd.) and single‐stranded cDNA libraries were prepared using Verso cDNA synthesis kit (Thermo Scientific). The splicing pattern of the WT and mutant *PDE1B* mini‐gene transcripts was assessed, following PCR amplification and Sanger sequencing.

### 2.4 Western Blot Analysis

An expression plasmid that contained the investigated *PDE1B* transcript (NM_000924) was created via Vector Builder (https://en.vectorbuilder.com/). The WT construct was used as a template for the creation of three investigated variants: c.133C>T, p.Gln45* (primer forward: 5′‐ GGTGAAGTAGTTGGAGAATGGGGAGATAAACATTG‐3′, primer reverse: 5′‐ CCAACTACTTCACCATGTAGCGCAGCAG‐3′). C.256C>T, p.Gln86* (primer forward: 5′‐ CGAGCTGTAGGAGCTGCGGTCAGATGC‐3′, primer reverse: 5′‐ GCTCCTACAGCTCGTCCTCCGTGTCCAAGATTTG‐3′). c.892delA, p.Ser298Alafs*6 (primer forward: 5′‐CCACATCGCTCTGTTTTCCGATTGATGCAGG‐3′, primer reverse: 5′‐CAGAGCGATGTGGTGATTCTCCAGCACTGAAC‐3′). All four plasmids were fully verified through Sanger sequencing. Each plasmid (750 ng) was co‐transfected with a 75 ng GFP‐tagged plasmid into HEK293 cells, at 60% confluence in 12‐well plates, using Polyjet transfection reagent (PolyJet™, SignaGen®, USA)). Twenty‐four hours post‐transfection, protein lysates were extracted from the cells using radioimmunoprecipitation assay (RIPA) buffer (50 mM tris–HCl [pH 8], 150 mM NaCl, 1% Nonidet P‐40, 0.5% sodium deoxycholate, 0.1% SDS, 1 mM dithiothreitol [DTT], and 1:100 protease inhibitor mixture [Sigma‐Aldrich]). The lysate was heated for 5 min at 95° and loaded onto sodium dodecyl‐sulfate polyacrylamide gel electrophoresis (SDS‐PAGE) (10% acrylamide). Blocking was done with phosphate‐buffered saline with 0.1% Tween 20 (PBST) mixed with 10% skimmed milk for 1 h. After blocking, membranes were incubated overnight at 4°C with primary antibodies: Anti‐Flag (1:1000, F1804, monoclonal produced in mouse; Sigma‐Aldrich) and Anti‐Actin (0869100‐CF; MP Biomedicals). The membranes were then washed three times with PBST for 5 min. Finally, the membranes were incubated with secondary antibody for 1 h at room temperature and washed with PBST (once for 15 min and twice for 5 min). Blots were developed using WesternBright Quantum HRP substrate chemiluminescence reagent (K‐12045‐D20; Advansta). To visualize the PDE1B protein in cell lysates transfected with various variant types, different exposure times were required. Exposure durations tested were 5 s, 10 s, 15 s, 30 s, and 1 min. The complete Western blot membranes at these different exposure times are shown in Figure S3.

## 3 Results

### 3.1 Clinical Studies and *PDE1B* Variants

**FIG. 1:**
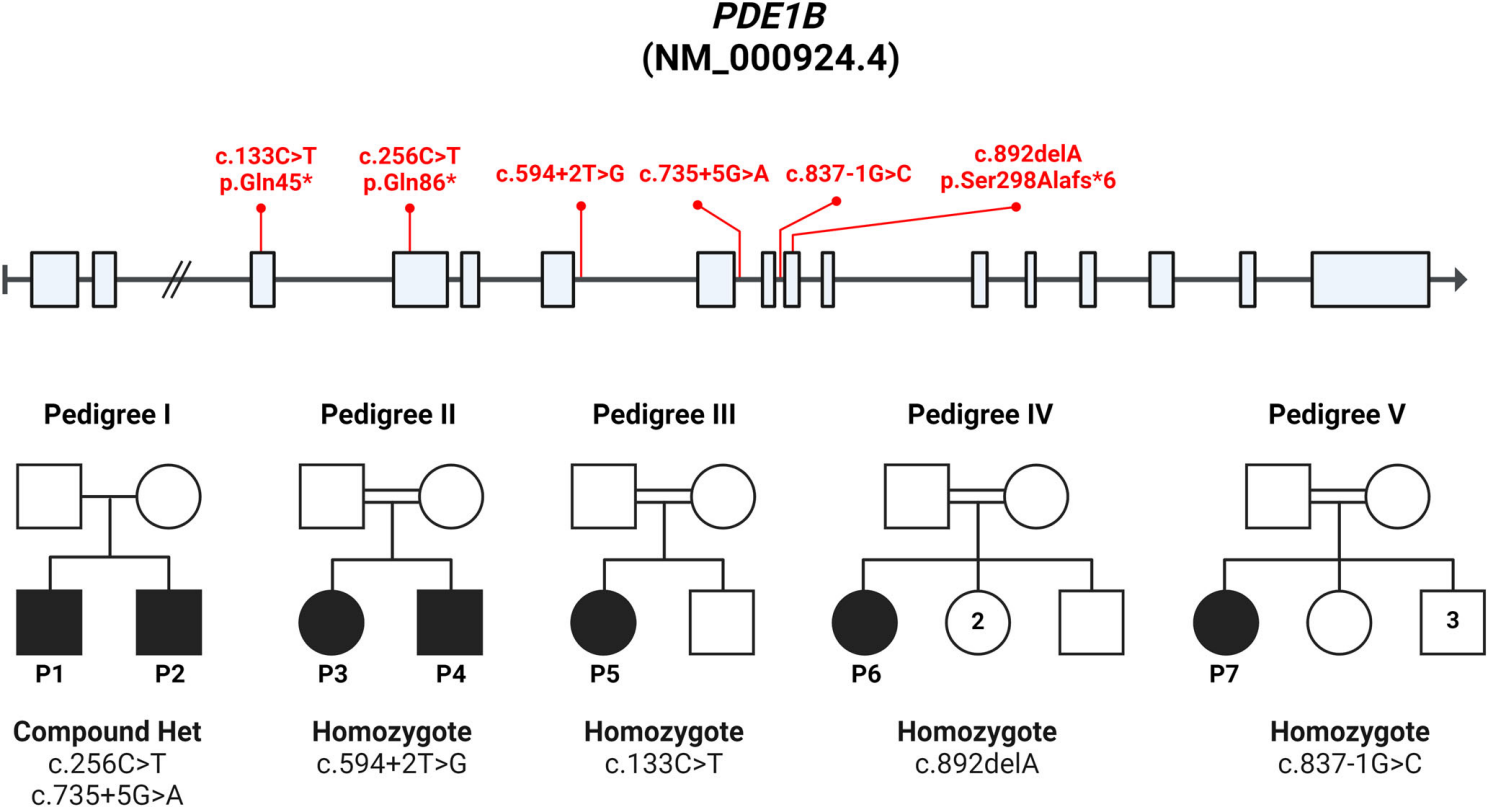
Identification of *PDE1B* variants in five different families. The genomic structure of *PDE1B* (NM_000924.4) is illustrated, with the locations of the investigated variants highlighted in red. Pedigrees of the five investigated families are shown. Affected individuals are filled in black and labeled P1–P7. Numbers inside the squares or circles represent the number of healthy siblings of the same gender. [Color figure can be viewed at wileyonlinelibrary.com]

Through GeneMatcher we identified seven affected individuals of five non‐related families with similar phenotypes (Table 1, detailed in Table S1), all with biallelic variants in *PDE1B* that segregated within their families as expected for autosomal recessive heredity (Fig. 1, Fig. S1). All seven individuals were born at term following uneventful pregnancies, had hypotonia (evident at birth or within the first few months of life), with later evolving dystonia/chorea/ataxia, motor delay (walking at 2–3 years of age), as well as significant speech delay and mild to moderate intellectual disability. Notably, fatigue with intermittent head drop forward was evident in some individuals. Birth weight was appropriate for gestational age (AGA) and there was no failure to thrive, no consistent facial dysmorphism, or other significant findings. There were no specific consistent findings on brain magnetic resonance imaging (MRI. The five pedigrees under investigation, and their associated variants, are shown in Figure 1. In more detail:

**TABLE 1:** Summary of the clinical findings of seven patients with *PDE1B* biallelic variants

Pedigree	Patient 1	Patient 2	Patient 3	Patient 4	Patient 5	Patient 6	Patient 7
	Pedigree I	Pedigree 1	Pedigree II	Pedigree II	Pedigree III	Pedigree IV	Pedigree V
Variant	Compound heterozygote	Compound heterozygote	Homozygote	Homozygote	Homozygote	Homozygote	Homozygote
Mutation type	Truncating + Splicing	Truncating + Splicing	Splicing	Splicing	Truncating	Frameshift	Splicing
cDNA mutation	c.256C>T, c.735 + 5G>A	c.256C>T, c.735 + 5G>A	c.594 + 2 T>G	c.594 + 2 T>G	c.133C>T	c.892delA	c.837‐1G>C
Consanguinity	No	No	Yes	Yes	Yes	Yes	Yes
Gender	M	M	F	M	F	F	F
Ethnic Group	N/A	N/A	South Asia	South Asia	Egyptian	Bedouin	Saudi Arabia
**Clinical findings**							
Hypotonia	N/A	N/A	Yes	Yes	Yes	N/A	No
Dystonia	N/A	N/A	Yes	Yes	Yes	Yes	Yes
Difficulties initiating movements	N/A	N/A	Yes	Yes	Yes	N/A	Yes
Head drop	N/A	N/A	Yes	Yes	Yes	N/A	N/A
Ataxia	Yes	N/A	Yes	Yes	Yes	N/A	Yes
Intellectual disability	Yes	N/A	Yes	Yes	Yes	N/A	Yes
Speech delay	Yes	N/A	Yes	Yes	Yes	N/A	Yes
Delayed motor development	Yes	N/A	Yes	Yes	Yes	Yes	Yes
Brain MRI	Normal	N/A	N/A	Normal	Thin CC	N/A	Normal

Abbreviations: M, male; F, female; N/A, not available; CC, corpus callosum; MRI, magnetic resonance imaging.

Family I: The only two sons of non‐consanguineous parents presented with a similar disease phenotype. Both were born at term with AGA birth weights, following uneventful pregnancies. Hypotonia was evident in early infancy with ataxia developing later, requiring the use of a walker at school. Both boys had speech delay and moderate intellectual disability. In terms of motor development, walking was achieved at 2 years of age. Brain computed tomography (CT) done for one of the brothers was normal. Both brothers were compound heterozygous for two *PDE1B* variants: a nonsense variant chr12:54,569,212C>T c.256C>T, p.(Q86*) and a non‐canonical splice site variant chr12:54,572,746G>A, c.735 + 5G>A.

Family II: Two affected individuals (one male and one female) aged 23 and 26 years, the only children of first cousins of South Asian ancestry, were born at term with AGA birth weights, following uneventful pregnancies. In both individuals, hypotonia was evident soon after birth, with later development of dystonia with difficulties in initiating movements. Both individuals have mild to moderate intellectual disability (mainstream schools with personalized support) and have dysarthria and speech difficulties, using only single words at adulthood. Motor development was also delayed, and both started walking at age 3 years. Notably, now into their 20s, both have fatigue after 10–15 min with intermittent head drop forward, possibly due to fatigue of neck muscles. Some truncal fatigue is also evident when sitting for prolonged periods and there is also mild facial weakness. Facial, bulbar, and limb movements are sluggish and have a dystonic element. There were no behavior abnormalities, failure to thrive, microcephaly, or dysmorphism. Both have myopia yet have normal eye movements. As adults, they live at home with their parents. They are independently mobile, but with poor balance. They can follow simple instructions and require assistance for all activities of daily living. MRI of brain and neck was normal. Nerve conduction studies and electromyography (EMG) were normal. Both affected individuals were homozygous for a *PDE1B* splice variant chr12:54,570,359 T>G, c.594 + 2 T.

Family III: A 5‐year‐old daughter of consanguineous parents of Egyptian ancestry was born at term (40 weeks), with AGA birth weight, following an uneventful pregnancy. Hypotonia was evident at birth, and dystonia in the left upper limb was first noted at 9 months with no response to levodopa. Additionally, she exhibited difficulties in initiating movements, similar to both patients in Pedigree II. She began walking at 3 years, but her gait remained mildly ataxic and unsteady with a wide base, accompanied by a flexed neck drop to one side. Intellectual disability and speech delay were evident, though by the age of 5 years she could understand and communicate verbally. Currently, she is in school, with an average IQ of 90. She can learn, obey commands, and respond well during examinations. Although she had speech delay, only starting to speak at 20 months, she now speaks well but with some dysarthric letters. She had borderline dysmorphic features, including a long face, high forehead, sparse thin eyebrows, depressed nasal bridge, wide‐spaced eyes, prominent broad nose, long philtrum, thin upper lip, everted lower lip, and large low‐set ears. She also has infrequent drooling. The phenotype is non‐progressive. Brain MRI revealed a thin corpus callosum, mild deep white matter signal changes on T2, and T‐FLAIR (fluid‐attenuated inversion recovery) around the occipital horn, with more pronounced effects on the right side. Her abdominal ultrasound, EMG, nerve conduction velocity (NCV), auditory brainstem response (ABR), echocardiography, metabolic workup, and karyotype were all normal, as were blood creatine phosphokinase (CPK) and liver function tests. Genetic testing unraveled homozygosity for the *PDE1B* variant chr12:54566993C>T, c.133C>T p.(Gln45Ter).

Family IV: A female child of consanguineous parents (double first cousins) of Bedouin ancestry, with three healthy siblings, was born at term, with AGA birth weight, following an uneventful pregnancy. Her developmental milestones were delayed: at 7 months she was unable to sit independently; at 1.5 years old she could roll over and stand with support. There was no dysmorphism. Unrelated to her neurological disorder, she had a severe infection at 1.6 years with secondary hypoxic–ischemic encephalopathy (HIE) and ischemic damage (evident on brain CT) and eventually died at age 3.5 years. Thus, it was difficult to determine the natural course of her hereditary neurological disorder beyond the age of 1.6 years. Whole exome sequencing identified a homozygous chr12:54573409C>A, c.892delA, p.(Ser298Alafs*6).

Pedigree V: A 20‐year‐old female born to consanguineous parents (first cousins) of Saudi Arabian descent, with five healthy siblings, was born at term. At age 3 years, she began showing signs of ataxia, gait abnormalities, and dystonia, which gradually worsened, leaving her reliant on assistance for daily activities such as feeding, dressing, and bathing. She struggles with initiating movements and exhibits head turning, along with twisting movements of her upper and lower limbs, accompanied by abnormal involuntary movements. Her speech development was delayed, with speech initiation occurring at age 3 years, and she continues to have dysarthric speech. She began walking at 18 months but experienced frequent falls. Additionally, she has poor school performance and has suffered from sleep disturbances since the age of 5 years. An MRI performed at age 17 years was normal. She also presents with short stature and S‐shaped scoliosis. The patient was found to be homozygous for the splicing variant chr12:54573354 G>C, c.837‐1G>C.

### 3.2 Splicing Impact of the Deleterious Intronic Variants

**FIG. 2:**
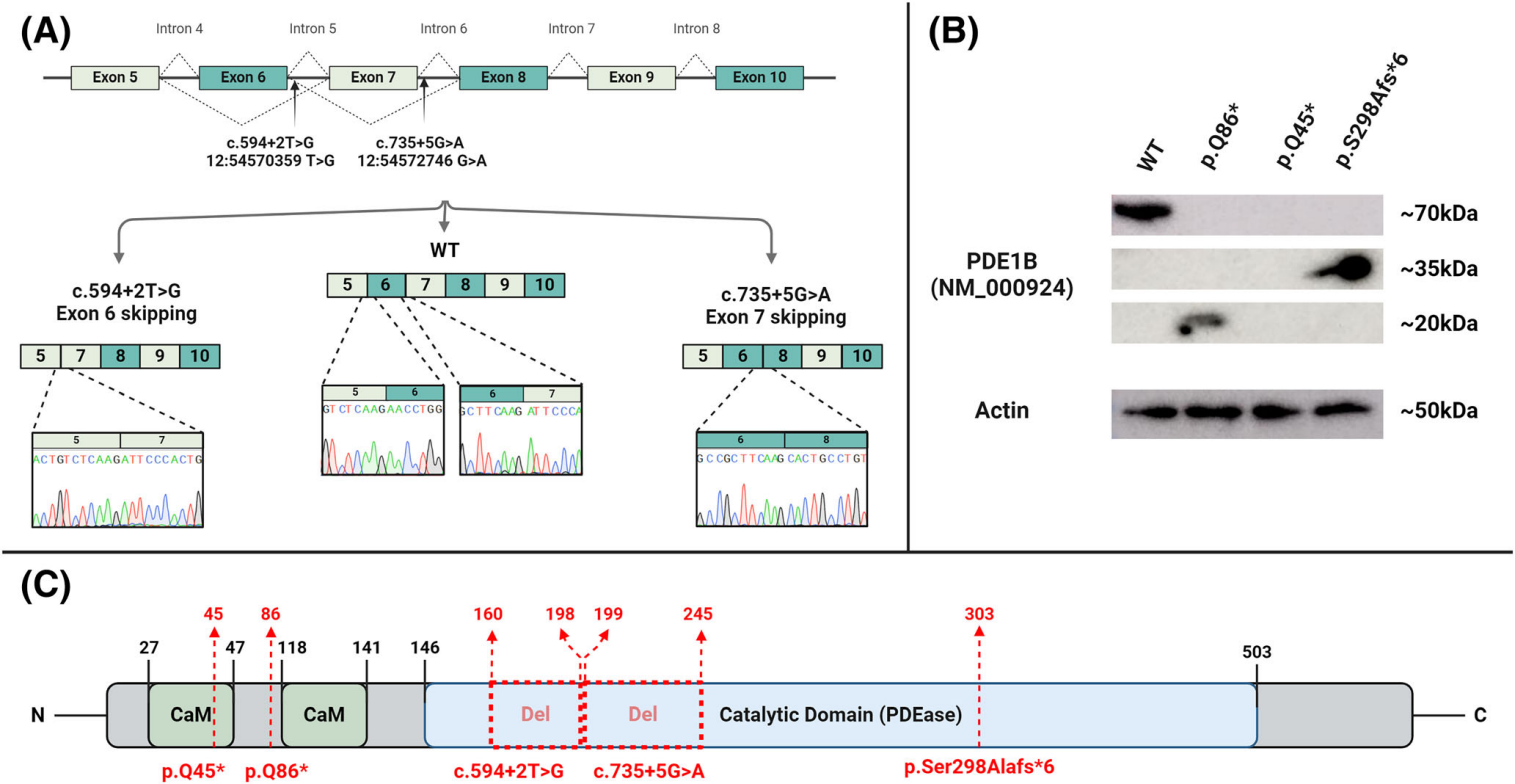
In vitro studies of the *PDE1B* variants. (A) Mini‐gene splicing assay results of two examined splicing variants. (B) Western blot analysis of three investigated variants. (C) Diagram of the PDE1B protein structure showing the calmodulin domain (green area) and the catalytic phosphodiesterase (PDEase) domain (blue area). The locations of these domains are specified with black numbers. Splicing‐affected deletion sites are indicated by red squares and stop codon mutation sites within the protein domains are indicated by red arrows. WT, wild‐type. [Color figure can be viewed at wileyonlinelibrary.com]

To elucidate the functional impact of the *PDE1B* intronic variants, a mini‐gene splicing assay was conducted. *PDE1B* mammalian expression vectors containing exons 5–10 and their introns, with either the WT sequence or one of the two investigated variants (c.594 + 2 T>G and c.735 + 5G>A), were generated. These constructs were transiently expressed in SH‐SY5Y cells. RNA extraction and reverse transcription (details in Methods) confirmed the normal splicing of the two exon junctions (exons 5–6 and 6–7) in the WT construct. In contrast, the investigated variants, c.594 + 2 T>G and c.735 + 5G>A, showed aberrant splicing, resulting in exon 6 skipping and exon 7 skipping, respectively. These results were confirmed by PCR amplification and Sanger sequencing (Fig. 2A).

### 3.3 Protein Truncation Impact of the Coding Variants

Four expression plasmids of *PDE1B* (NM_000924), each flag‐tagged, were used to investigate the effect of coding variants on protein expression. These plasmids included the WT and three coding variants: p.Q45*, p.Q86*, and p.S298Afs*6. The plasmids were transfected into HEK293 cells, whose protein lysates were subsequently extracted. The WT plasmid produced the expected 70 kDa protein. In contrast, the plasmids harboring the p.S298Afs*6 and p.Q86* variants generated aberrant shorter proteins of 35 kDa and 20 kDa, respectively, with no normal‐sized protein detected. The p.Q45* variant did not produce any detectable protein (Fig. 2B). Notably, the WT PDE1B protein band was detectable at a low exposure time of 5 s. In contrast, the two aberrant proteins, p.S298Afs*6 and p.Q86*, were evident only at higher exposure times of 30 s and 1 min, respectively. The earliest truncating variant, p.Q45*, did not show any protein even at a high exposure time of 1 min. The Western blots at these different exposure times are shown in Figure S2.

## 4 Discussion

We describe a novel movement disorder syndrome caused by biallelic variants in *PDE1B*. We have thoroughly characterized the phenotypes of seven affected patients of four unrelated families and have molecularly confirmed the effects of six distinct variants: three splicing variants and three loss‐of‐function variants (two stop‐gain variants, and one indel that results in a deleterious frameshift).

The PDE superfamily consists of 11 subtypes (PDE1–PDE11), classified primarily based on sequence homology and encoded by 21 identified genes.^1^ Most of these subtypes have multiple gene products. PDEs are enzymes that uniquely terminate cyclic nucleotide signaling by catalyzing the hydrolysis of cAMP and cGMP. They are crucial regulators of the intracellular concentrations of cAMP and cGMP, as well as their signaling pathways and downstream biological effects.^24^ The PDEs are known to have many roles in the central nervous system (CNS),^25^ most notably in the basal ganglia that are critical in regulating motor control.^26^ PDE1B and PDE10A are the most prevalent PDEs in the basal ganglia, with both being equally present in the caudate nucleus.^27^ PDE1B mRNA levels in the caudate nucleus are 10 to 100 times higher than those of PDE1C and PDE1A, respectively.^27^

Dysfunction in the basal ganglia circuit, with the involvement of PDEs, has been implicated in various CNS disorders, including PD and HD. Notably, studies in both animal models and patients suggest an impairment of cyclic nucleotide signaling in HD.^28^ PDE10A is the phosphodiesterase most implicated in HD due to its high concentration in the striatum.^27^, ^28^ However, there is also evidence suggesting that other PDEs, such as PDE1B, may play a role in the pathogenesis of HD.^5^, ^29^, ^30^, ^31^ PDE1B mRNA levels have been reported to be reduced in transgenic mouse models of HD, including R6/2, R6/1, and N171‐82Q,^5^, ^32^, ^33^, ^34^ as well as in cDNA derived from mRNA of symptomatic HD patients.^5^, ^35^ Furthermore, the PDE1 inhibitor vinpocetine has been reported to improve both biochemical and behavioral abnormalities in toxic models of HD.^1^ Moreover, there are suggestions that cyclic nucleotide signaling might also be altered in PD. Evidence shows that in the 6‐hydroxydopamine (6‐OHDA) Parkinson's animal model, there is a reduction in cGMP levels in the striatum and globus pallidus, accompanied by an increase in striatal cAMP levels. These alterations may be associated with the activity of PDE1B or PDE10A.^5^, ^36^, ^37^, ^38^, ^39^

Recent findings that variants in genes encoding various PDEs cause rare forms of monogenic parkinsonism and chorea highlight the crucial role of PDEs in the pathophysiology of different movement disorders. *PDE2A* homozygous variants have been confirmed to cause a complex infantile syndrome consisting of childhood‐onset chorea, paroxysmal dyskinesia with cognitive disability, and electroencephalographic abnormalities or overt epilepsy.^11^, ^12^ It has been suggested that the high expression of PDE2A in extra‐striatal brain regions might account for the presence of additional neurological signs, beyond chorea. Heterozygous variants in *PDE8B* have been shown to underlie autosomal dominant striatal degeneration (ADSD),^8^, ^9^, ^10^ a rare genetic disorder marked by slowly progressive dysarthria, brisk deep tendon reflexes, and mild parkinsonism without tremor, along with a poor response to L‐dopa treatment. ADSD typically begins in the fourth to fifth decade of life and is distinguished by a unique MRI pattern showing symmetrical hyperintensities in the caudate, putamen, and nucleus accumbens. In all cases, heterozygous frameshift variants in *PDE8B* were reported to create a stop codon, resulting in a severely truncated and nonfunctional protein.^8^, ^9^, ^10^ Heterozygous *PDE10A* variants have been associated with a childhood‐onset, non‐progressive chorea syndrome that features bilateral striatal hyperintensities on MRI^14^, ^16^, ^17^, ^18^: Biallelic loss‐of‐function *PDE10A* variants, which cause significant PDE10A loss in the striatum, were shown to cause an infantile‐onset hyperkinetic chorea movement disorder.^15^ Interestingly, patients with these biallelic variants do not display the pronounced striatal abnormalities seen in imaging studies of heterozygous patients, indicating that distinct pathogenic mechanisms might be involved.

The PDEs most abundant in the basal ganglia are PDE10A and PDE1B. We now delineate a novel syndrome caused by biallelic variants in *PDE1B* (NM_000924.4), encoding phosphodiesterase 1B. We investigated through molecular studies the six disease‐causing variants found in the seven affected individuals: two splicing variants (c.594 + 2 T>G, c.735 + 5G>A), two truncating variants (c.133C>T, c.256C>T), and one small deletion with deleterious frameshift (c.892delA). The c.594 + 2 T>G splicing variant has been molecularly confirmed to cause skipping of exon 6, which is 117 base pairs in length, resulting in deletion of 39 amino acids (from positions 160 to 198). Similarly, the c.735 + 5G>A variant leads to skipping of exon 7, which is 141 base pairs long, resulting in deletion of 47 amino acids (from positions 199 to 245; Fig. 2A). Both exons are in‐frame, so their skipping does not cause a frameshift or a premature stop codon; however, these deletions occur within the catalytic domain of the protein, likely disrupting its function (Fig. 2C). As we show through protein studies in transfected HEK293 cells, the c.892delA (p.S298Afs*6) deletion variant generates a premature stop codon that disrupts the catalytic domain of the PDE1B protein, resulting in an aberrant 35 kDa protein; the c.256C>T (p.Q86*) truncating variant occurs upstream of the catalytic domain, also disrupting the functional calmodulin (CaM) domain, producing an aberrant 20 kDa protein. The most upstream deleterious variant, c.133C>T (p.Q45*), leads to the complete absence of any detectable protein (Fig. 2B, S2A,B). Moreover, it is important to note that detecting the aberrant proteins through Western blotting required more than six times the exposure time compared with that needed for detection of the WT protein (Fig. S2). This suggests a lower amount of protein and likely indicates that the aberrant transcripts are being disrupted at the mRNA level through nonsense‐mediated decay (NMD).

Analysis of the Genome Aggregation Database (GnomAD v4.1.0) revealed 44 potential loss‐of‐function (LOF) variants within *PDE1B* across over 750,000 individuals from various healthy populations (Table S2). Notably, 43 of these variants were not observed in a homozygous state in any individuals. However, one variant (GRCh38 12:54561606C>T, p.Gln8*) was identified in a homozygous state in three individuals. This exception is likely due to the variant being located in a coding region specific to only one of the nine PDE1B isoforms (Fig. S3), suggesting redundancy of this isoform. Notably, none of the *PDE1B* variants in the affected individuals we describe affect specifically this isoform (Fig. S3).

The PDE1B enzyme is part of the PDE1 family, known to be Ca2^+^/calmodulin‐dependent. Calmodulin (CaM) is an intracellular protein that binds calcium to form the Ca2^+^/calmodulin complex, which interacts with numerous target proteins and peptides to regulate vital biological processes. This complex is essential for converting the PDE1B protein from an inactive state to its active conformation.^40^ The protein structure of PDE1B, as demonstrated via Uniprot,^41^ includes two CaM binding sites, allowing Ca2^+^/calmodulin to interact with and activate the enzyme, as well as a core catalytic PDEase domain, responsible for the hydrolysis of cAMP and cGMP^42^ (Fig. 2C). The catalytic domain is conserved across the PDE1 family and other PDE families.^43^ Our data demonstrate that all the studied variants disrupt the integral catalytic domain of the PDE1B protein: the two splicing variants lead to deletions within the conserved catalytic core (Fig. 2C); the deletion variant, p.S298Afs*6, results in a premature stop codon within this domain; and the stop codons created by the two other variants (p.Q45 and p.Q86*) are located upstream of the catalytic core, resulting in its complete absence.

The patients under investigation exhibited a diverse range of neurological and developmental features, though no clear genotype–phenotype correlation was identified. Shared findings included neonatal hypotonia and dystonia, which varied in its presentation among patients. Some displayed generalized dystonic movements, while others experienced dystonia localized to specific areas, such as the neck, around the mouth, or in the form of dystonic posturing of the arms or upper limbs. Patients also faced difficulties initiating voluntary movements, occasionally accompanied by elements of chorea. Additional symptoms included neck drop and facial weakness. Ataxia was observed in varying degrees of severity, with some individuals requiring the use of a walker or wheelchair, while others could move independently but experienced frequent falls due to instability. All patients exhibited learning difficulties and mild to moderate intellectual disability. Motor developmental delay was universal among the patients, as was speech delay, with some also showing signs of dysarthria, which impaired their ability to articulate words clearly. The mild borderline dysmorphism described in a single patient (Pedigree III) is likely not specific to the syndrome.

PDE1B knockout (KO) mice on a C57BL/6N background provide additional evidence for the role of PDE1B in striatal function: studies have shown that these mice exhibit increased striatal dopamine turnover, which is linked to heightened baseline motor activity, and an exaggerated locomotor response to dopaminergic stimulants like methamphetamine and amphetamine.^44^, ^45^ Moreover, *Pde1b*‐KO mice displayed spatial learning deficits^45^ and antidepressant‐like phenotypes.^39^

Altogether, we have identified and characterized a novel monogenic movement disorder caused by biallelic *PDE1B* variants. Our findings are in line with increasing evidence that PDEs play a role in both rare and common movement disorders, largely due to their involvement in regulating cyclic nucleotide signaling. Thus, advances in developing treatment modalities for common hyperkinetic and hypokinetic movement disorders, such as HD and PD,^1^, ^5^ might be conducive to achieving effective treatment of the rare *PDE1B* disease, and vice versa.

## Author Roles

Study initiation: T.P., V.H., H.H., S.M., R.M., O.S.B. Clinical studies: E.K., V.H., E.G.S., S.A., A.N., M.L.T., L.A.A., S.M., H.H., O.S.B. Genetic studies: T.P., N.H., I.A., N.Y.R., J.A.P., Y.A., L.A., S.M., R.M., M.L.T., L.A.A., S.M., H.H., O.S.B. Molecular studies: T.P., V.D., A.S., N.A., M.J., O.F., O.S.B. Writing of the manuscript: T.P. and O.S.B. with contributions and edits of all authors. All authors read and approved the final manuscript.

## Supporting information


**Figure S1.** Segregation of the variants as determined by Sanger sequencing.

**Figure S2.** Western blot (WB) analysis of the investigated variants. (A) WB showing the wild‐type (WT) and the three other investigated variants at different exposure times (5, 10, and 15 s). The analysis was performed using two different antibodies: Anti‐Flag and Anti‐Actin. (B) The WT protein was excised from the membrane. An exposure time of 1 min was used.

**Figure S3.** Distribution of *PDE1B* variants. All six investigated variants are shown in black; the variant found in homozygous healthy individuals per gnomAD is highlighted in red.

**Table S1.** Detailed clinical findings of the patients with *PDE1B* biallelic variants.

**Table S2.** Analysis of the Genome Aggregation Database (GnomAD v4.1.0) revealed 44 potential loss‐of‐function (LOF) variants within *PDE1B* across over 750,000 individuals from various healthy populations.

## Data Availability

The data that support the findings of this study are available on request from the corresponding author. The data are not publicly available due to privacy or ethical restrictions.
