# Supplementary figures and images for: Early‐Onset Movement Disorder Syndrome Caused by Biallelic Variants in PDE1B Encoding Phosphodiesterase 1B
Source: Mov Disord. 2025 Jun 10;40(9):1874–82. doi: 10.1002/mds.30249 (PMC12485581; doi:10.1002/mds.30249)

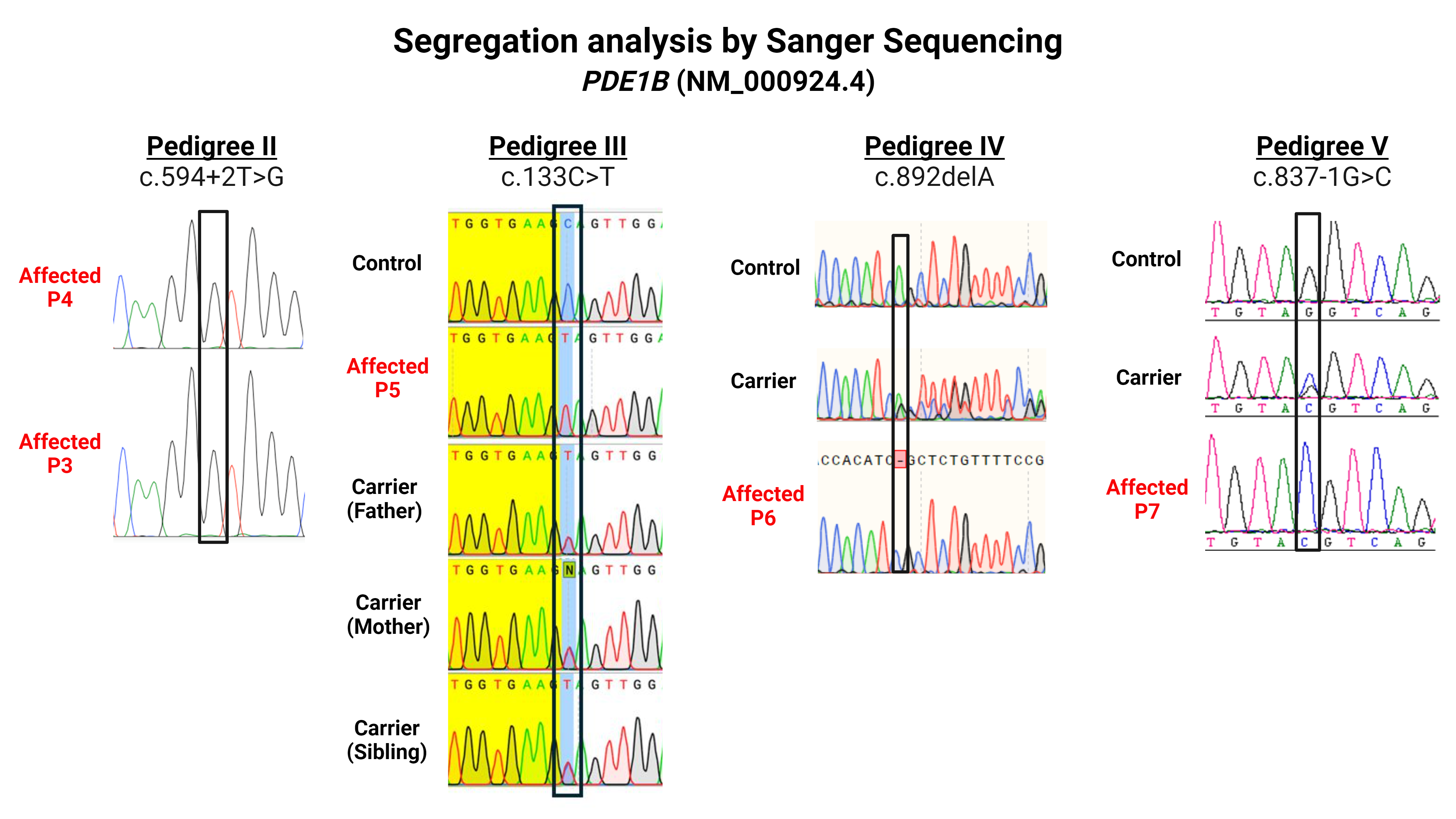

Supplement: Supplementary file 1 — Figure S1. Segregation of the variants as determined by Sanger sequencing. [file MDS-40-1874-s004.png]

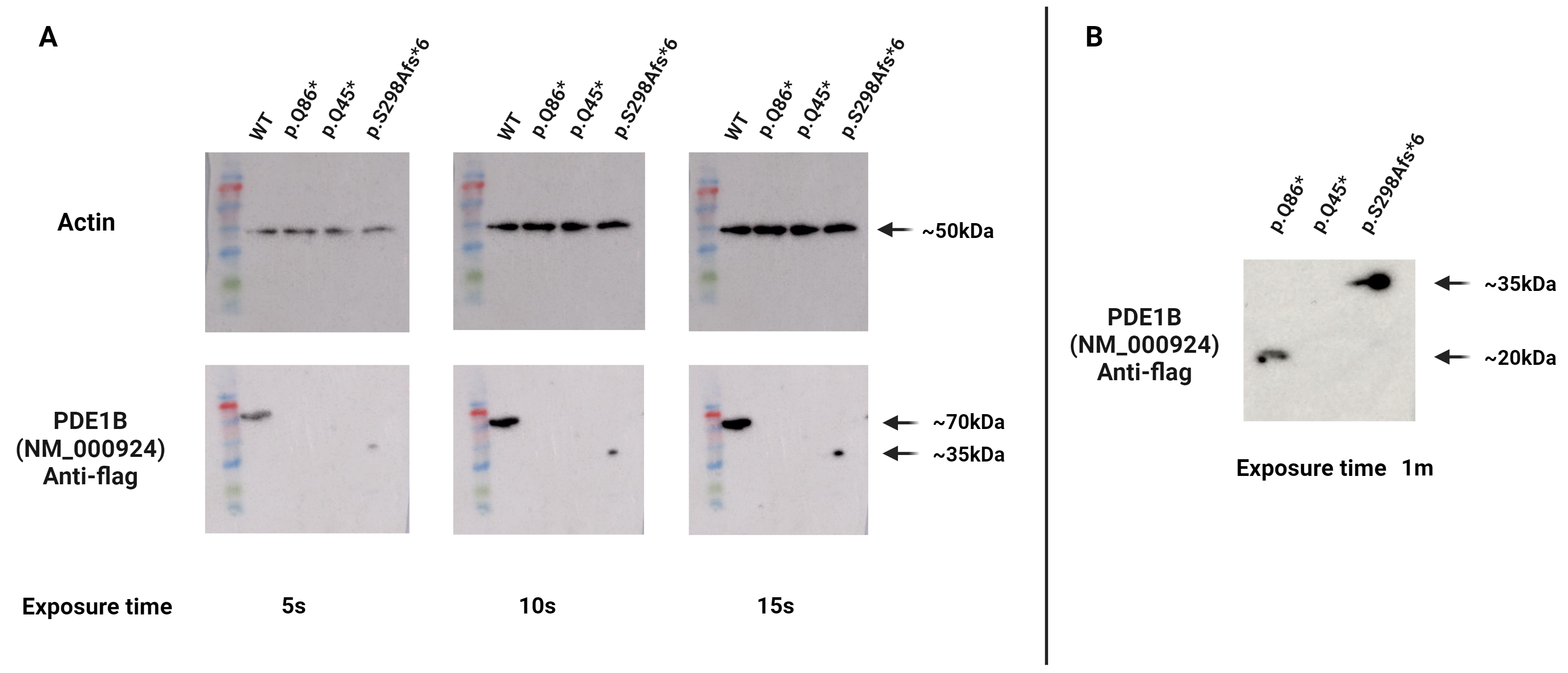

Supplement: Supplementary file 2 — Figure S2. Western blot (WB) analysis of the investigated variants. (A) WB showing the wild‐type (WT) and the three other investigated variants at different exposure times (5, 10, and 15 s). The analysis was performed using two different antibodies: Anti‐Flag and Anti‐Actin. (B) The WT protein was excised from the membrane. An exposure time of 1 min was used. [file MDS-40-1874-s002.png]

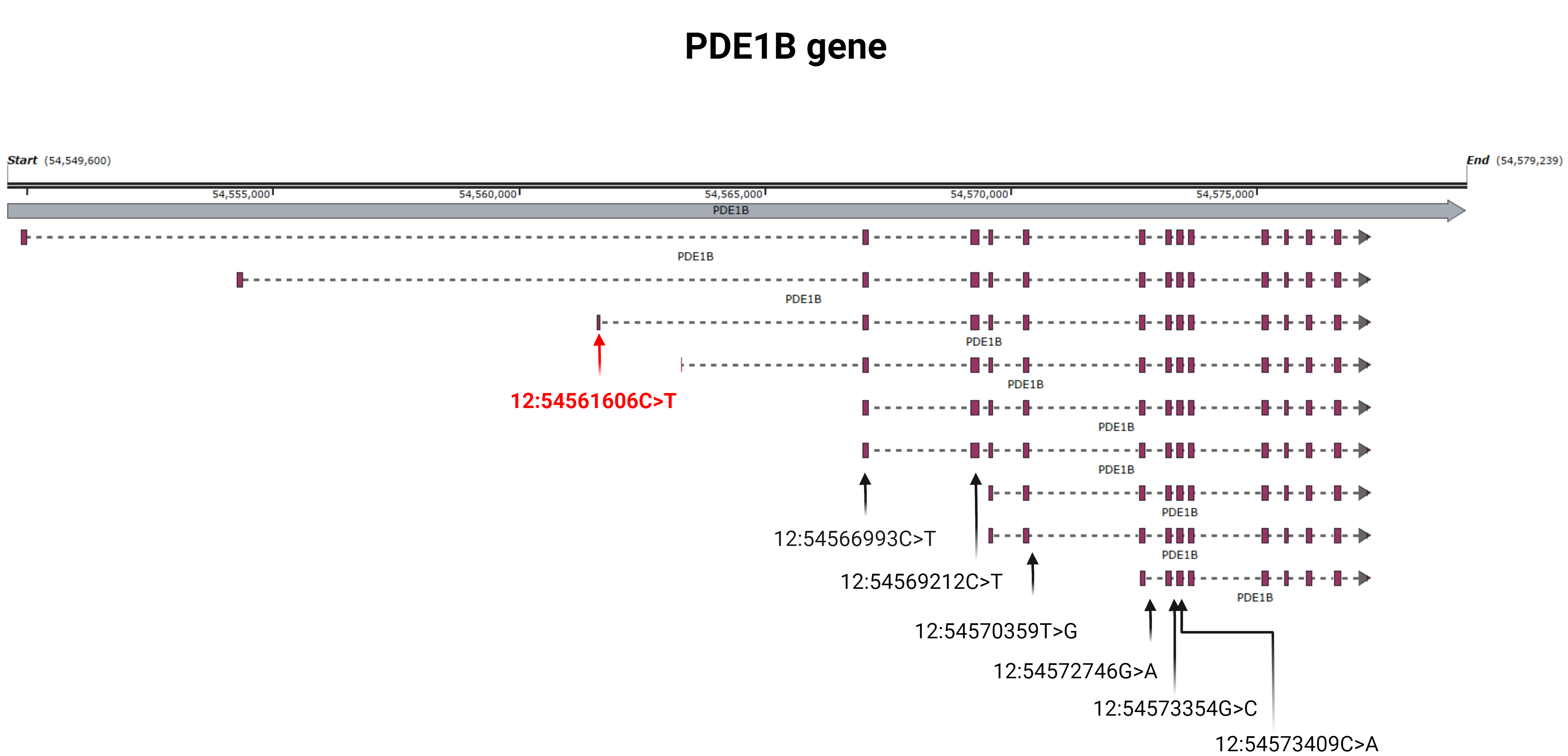

Supplement: Supplementary file 3 — Figure S3. Distribution of PDE1B variants. All six investigated variants are shown in black; the variant found in homozygous healthy individuals per gnomAD is highlighted in red. [file MDS-40-1874-s005.png]
